# Design and Simulation of A Novel Piezoelectric AlN-Si Cantilever Gyroscope

**DOI:** 10.3390/mi9020081

**Published:** 2018-02-15

**Authors:** Jian Yang, Chaowei Si, Fan Yang, Guowei Han, Jin Ning, Fuhua Yang, Xiaodong Wang

**Affiliations:** 1 Institute of Semiconductors, Chinese Academy of Sciences, Beijing 100083, China; yangjian@semi.ac.cn (J.Y.); yangfan3104@semi.ac.cn (F.Y.); hangw1984@semi.ac.cn (G.H.); ningjin@semi.ac.cn (J.N.); fhyang@semi.ac.cn (F.Y.); 2 College of Materials Science and Opto-Electronic Technology, University of Chinese Academy of Sciences, Beijing 100049, China; 3 State Key Laboratory of Transducer Technology, Chinese Academy of Sciences, Beijing 100083, China; 4 School of Electronic, Electrical and Communication Engineering, University of Chinese Academy of Sciences, Beijing 100049, China; 5 School of Microelectronics, University of Chinese Academy of Sciences, Beijing 100049, China

**Keywords:** microelectromechanical systems (MEMS), aluminum nitride, piezoelectric effect, cantilever, gyroscope, simulation

## Abstract

A novel design of piezoelectric aluminum nitride (AlN)-Si composite cantilever gyroscope is proposed in this paper. The cantilever is stimulated to oscillate in plane by two inverse voltages which are applied on the two paralleled drive electrodes, respectively. The whole working principles are deduced, which based on the piezoelectric equation and elastic vibration equation. In this work, a cantilever gyroscope has been simulated and optimized by COMSOL Multiphysics 5.2a. The drive mode frequency is 87.422 kHz, and the sense mode frequency is 87.414 kHz. The theoretical sensitivity of this gyroscope is 0.145 pm/◦/s. This gyroscope has a small size and simple structure. It will be a better choice for the consumer electronics.

## 1. Introduction

Microelectromechanical systems (MEMS) gyroscopes are core inertial sensors. They are widely used in smartphones, unmanned aerial vehicles (UAV), automotive electronics, or other consumer goods [1]. In recent years, aluminum nitride (AlN) piezoelectric gyroscopes become a new research trend. The AlN gyroscopes can be categorized into two main types: (1) thin-film AlN gyroscopes and (2) bulk acoustic wave (BAW) AlN-on-Si gyroscopes. The thin-film AlN gyroscopes have traditional structures: beams and proof masses. The AlN thin-film gyroscope relies on bending vibration of beams. The drive mode is simulated by voltages and the Coriolis force is detected through piezoelectric charge. While, the BAW AlN-on-Si gyroscopes are made up of an AlN film on silicon substrate with a specific geometry. Two degenerate bulk acoustic wave modes of the AlN film, which are orthogonal to each other, are used as the drive mode and sense mode, respectively.

The Albert P. Pisano’s group, which comes from the University of California, focuses on the thin-film AlN gyroscopes [2,3,4]. The structure contains three layers: the AlN layer and the bottom/top electrode layers. The thickness of AlN is 2 μm. These gyroscopes have a high efficiency of electromechanical transduction. However, a near zero stress AlN film is needed. It is difficult for the AlN deposition process, and the robustness of this gyroscope is another serious problem. Farrokh Ayazi’s group, which is from the Georgia Institute of Technology, focuses on the BAW AlN-on-Si gyroscopes [5,6,7]. From 2013 to the present, many different kinds of BAW gyroscopes have been researched and optimized. These gyroscopes have a relatively high frequency (3.1–11 MHz) and perfect immunity to shock and vibration. However, a high frequency will lead to a reduction of response amplitude. The sensitivity will decrease. Besides, the size of these BAW gyroscopes are very large. 

Considering the characteristics of the two AlN gyroscopes, a novel AlN-Si composite cantilever gyroscope is proposed in this paper. Based on the piezoelectric effect of AlN, the cantilever will be excited to bend in-plane. The working frequency is 87 kHz, which is lower than the frequency of BAW AlN-on-Si gyroscopes. This will benefit the sensitivity. This gyroscope has a small size and simple structure. Analysis and simulation of this piezoelectric gyroscope has been carried out and reported on in this paper. 

## 2. Working Principles

### 2.1. Working Principles of Drive Mode

**Figure 1:**
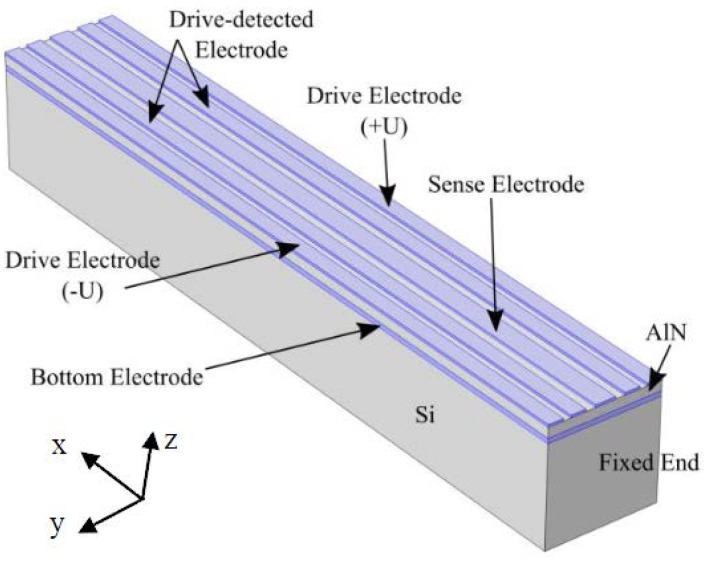
The structure of aluminum nitride (AlN)-Si composite cantilever gyroscope.

**Figure 2:**
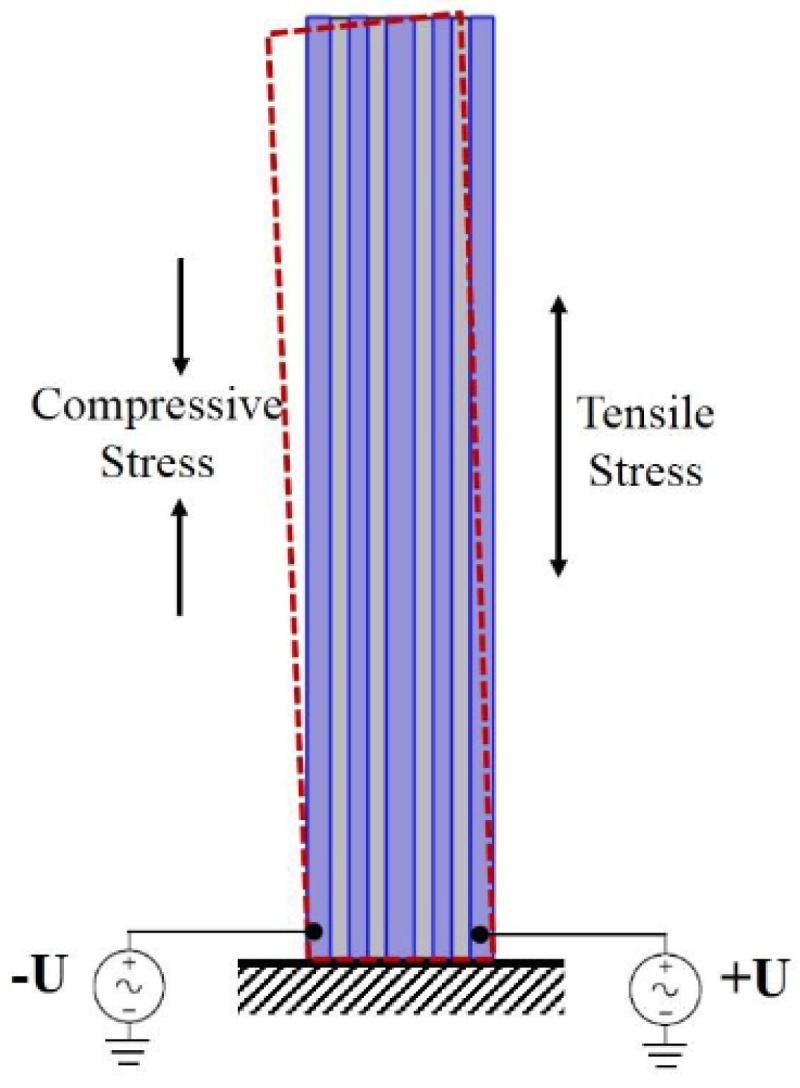
The schematic of in-plane vibration of cantilever.

The schematic of AlN-Si composite cantilever gyroscope is shown in Figure 1. It contains four layers. The bottom electrode layer is Mo, connecting the ground in electric field. The top layer is Al, divided into five parts. The five electrodes include two drive electrodes, two drive-detected electrodes, and one sense electrode. In this gyroscope, AlN layer works as drive function for the composite cantilever. Inverse voltages are applied on two drive electrodes respectively, as shown in Figure 1 and Figure 2. The drive-detected electrodes are used to detect the drive signal. The purpose is to stabilize both magnitude and frequency of the drive signal. Based on the piezoelectric effect of AlN, the two inverse voltages will excite two inverse stresses (±σ)—the compressive stress and tensile stress. The direction of the stresses are along with the length of cantilever. They will form a couple stress. The formula of *σ* has been deduced as shown in Equation (1).

(1) $$\begin{array}{c} \sigma =E_{\mathrm{AlN}}\frac{U}{d_{\mathrm{AlN}}}d_{31}=\frac{E_{\mathrm{AlN}}U_{0}\sin(\omega t)d_{31}}{d_{\mathrm{AlN}}}\\ U=U_{0}\sin(\omega t) \end{array}$$

where *E*_AlN_ is the Young’s modulus of AlN, *d*_AlN_ is the thickness of AlN, *U* is the sinusoidal drive voltage, *ω* is the angular frequency of drive voltage, and *d*_31_ is the piezoelectric coefficient of AlN. According to the references, *d*_31_ = −2.6 pm/V [8,9].

This couple stress will drive the AlN-Si composite cantilever to bend in-plane, as shown in Figure 2. The bending moment *M* is given by Equation (2). Where *W* represents the width of cantilever and *W*_dri_ represents the width of drive electrodes.

(2) $$\begin{array}{cc} M & =\sigma W_{\mathrm{dri}}d_{\mathrm{AlN}}(W-W_{\mathrm{dri}})\\ & =E_{\mathrm{AlN}}U_{0}\sin(\omega t)d_{31}W_{\mathrm{dri}}(W-W_{\mathrm{dri}}) \end{array}$$

The deformation of cantilever satisfies the approximately differential equation of flexural curve of Equation (3). By solving the Equation (3), the displacement function *y*(*x*,*t*) can be deduced as shown in Equation (4). Where EI_y_ denotes the flexural rigidity of the composite cantilever in y direction. *E*_Al_, *E*_Mo_, and *E*_Si_ are the Young’s modulus of Al, Mo, and Si respectively; and *d*_Al_, *d*_Mo_, and *d*_Si_ are the thickness of Al, Mo, and Si respectively. The *x* axial is parallel to the length direction of cantilever. *x* is the position, belonging to [0,L]. All the parameters are shown in Table 1.

(3) $$y^{\prime \prime }=\frac{M}{EI_{y}}$$

(4) $$y(x,t)=\frac{6E_{\mathrm{AlN}}d_{31}W_{\mathrm{dri}}(W-W_{\mathrm{dri}})}{W^{3}(E_{\mathrm{Al}}d_{\mathrm{Al}}+E_{\mathrm{AlN}}d_{\mathrm{AlN}}+E_{\mathrm{Mo}}d_{\mathrm{Mo}}+E_{\mathrm{Si}}d_{\mathrm{Si}})}x^{2}U_{0}\sin(\omega t)$$

**Figure 3:**
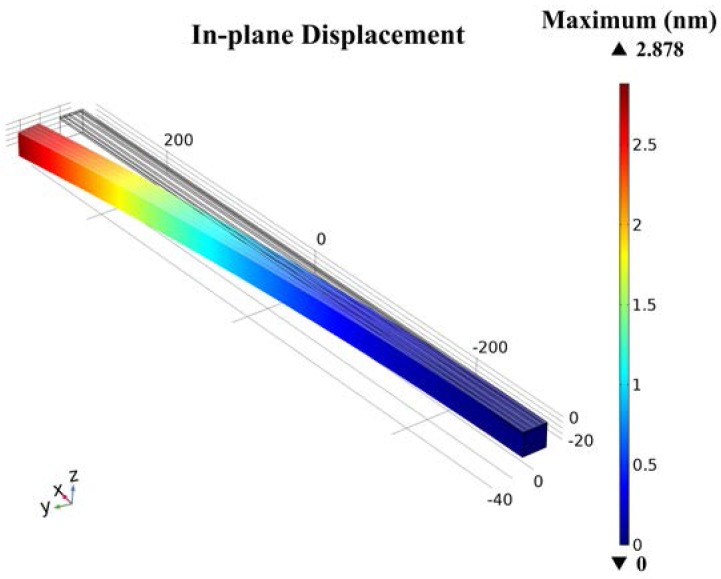
Displacement simulation of AlN-Si composite cantilever under ±1 V drive voltages, stationary study.

**Figure 4:**
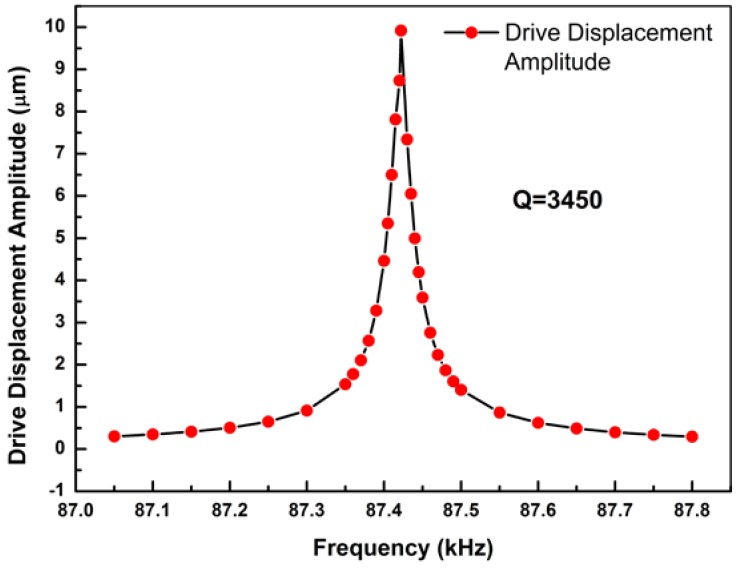
The resonant displacement amplitude of drive mode, frequency domain study.

Plugging the values into Equation (4), the theoretical value of maximum displacement *y*_max_ = *y*(*L*,$\frac{2n+1}{2\omega }\pi$) = 2.920 nm can be calculated. Meanwhile, the same cantilever model has been designed and simulated by COMSOL Multiphysics 5.2a (COMSOL Inc., Stockholm, Sweden). A stationary study has been simulated. The maximum stationary displacement of simulation value is *y*_s-max_ = 2.878 nm, as shown in Figure 3. Comparing the theoretical value to the simulation one, an error ratio 1.5% can be calculated. This proves that the deduction of working principles is logical and accurate. To further research the resonant properties, a frequency domain study has been done. The maximum resonant displacement is 9.92 μm, and the quality factor is 3450, as shown in Figure 4.

### 2.2. Working Principles of Sense Mode

This gyroscope can detect an angular rate which is along the length direction of cantilever. When an x axial angular rate Ω is applied, the cantilever will be driven to bend out-plane by Coriolis force *F_C_*(*x*,*t*). The *F_C_*(*x*,*t*) formula can be deduced as shown in Equation (5). The *ρ*_all_ is a linear density of the composite cantilever. *C*_0_ represents the constant coefficient of *y*(*x*,*t*). The direction of *F_C_*(*x*,*t*) is *z*-axial.

(5) $$\begin{array}{l} F_{C}(x,t)=2m(x)\Omega \overset{\cdot }{y}(x,t)\\ =\int_{0}^{x}2\rho_{\mathrm{all}}\Omega \overset{\cdot }{y}(x_{0},t)dx_{0}\\ =\frac{2}{3}\rho_{\mathrm{all}}\Omega \omega C_{0}\cos(\omega t)x^{3} \end{array}$$

$$\rho_{\mathrm{all}}=\frac{\rho_{\mathrm{Al}}V_{\mathrm{Al}}+\rho_{\mathrm{AlN}}V_{\mathrm{AlN}}+\rho_{\mathrm{Mo}}V_{\mathrm{Mo}}+\rho_{\mathrm{Si}}V_{\mathrm{Si}}}{L}$$

$$C_{0}=\frac{6E_{\mathrm{AlN}}d_{31}W_{\mathrm{dri}}(W-W_{\mathrm{dri}})}{W^{3}(E_{\mathrm{Al}}d_{\mathrm{Al}}+E_{\mathrm{AlN}}d_{\mathrm{AlN}}+E_{\mathrm{Mo}}d_{\mathrm{Mo}}+E_{\mathrm{Si}}d_{\mathrm{Si}})}U_{0}$$

By solving the approximately differential equation of flexural curve in *z* direction-Equation (6), a displacement function *z*(*x*,*t*) can be deduced. The function *z*(*x*,*t*) is shown in Equation (7). *EI_z_* is the flexural rigidity of the composite cantilever in *z* direction. The function *z*(*x*,*t*) is the displacement of sense mode. Therefore, a linear relation between *z* and Ω can be gotten from Equation (7). The maximum coefficient of *z*(Ω) is the sensitivity of this gyroscope.

(6) $$z^{\prime \prime }=\frac{M_{z}(x,t)}{EI_{z}}$$

$$M_{z}(x,t)=\int_{x}^{L}F_{C}(x_{0},t)\cdot (L-x_{0})dx_{0}$$

(7) $$z(x,t)=\frac{\rho_{\mathrm{all}}\omega C_{0}\Omega }{15EI_{z}}(\frac{1}{4}L^{5}x^{2}-\frac{1}{12}Lx^{6}+\frac{1}{21}x^{7})\cos(\omega t)$$

## 3. Design of AlN-Si Composite Cantilever Gyroscope

**Figure 5:**
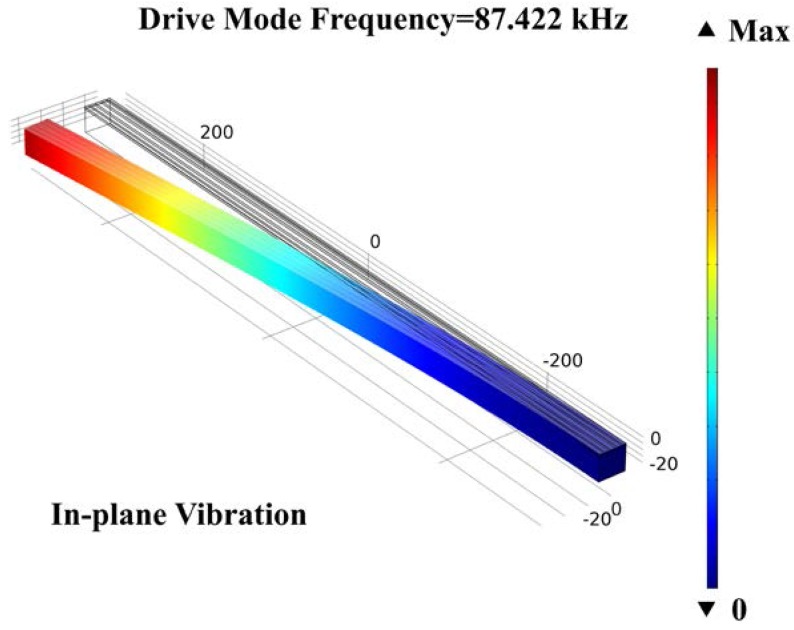
The drive mode simulation of the cantilever gyroscope.

**Figure 6:**
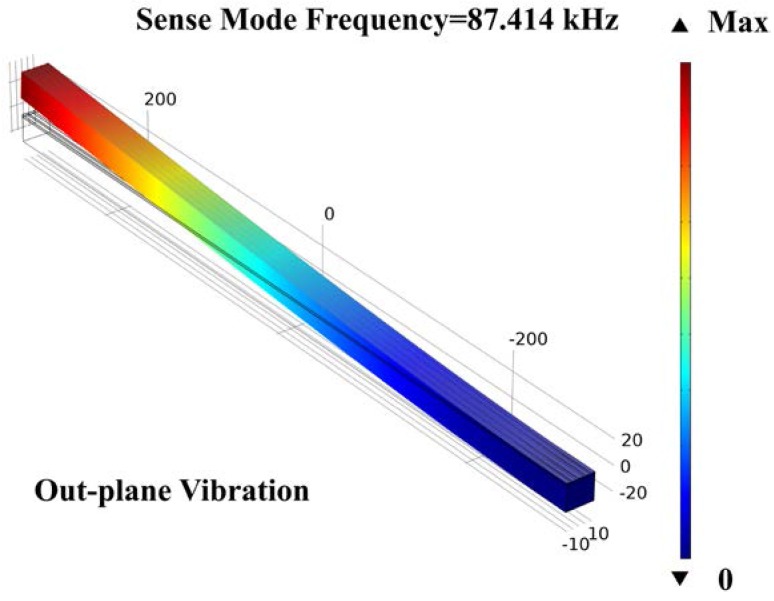
The sense mode simulation of the cantilever gyroscope.

The main structure of this gyroscope is shown in Figure 1. The sizes of AlN-Si composite cantilever are designed and optimized by COMSOL Multiphysics. To realize the mode matching, the parametric sweep function was used. The valve of the cantilever width has been swept from 20 μm to 25 μm, with a step of 0.01 μm. When the value is 22.87 μm, the frequencies of drive mode and sense mode are nearly equal. All the values are shown in Table 2. The simulation result of drive mode frequency is 87.422 kHz, and the sense mode frequency is 87.414 kHz. The mode shapes are shown in Figure 5 and Figure 6, respectively. 

**Figure 7:**
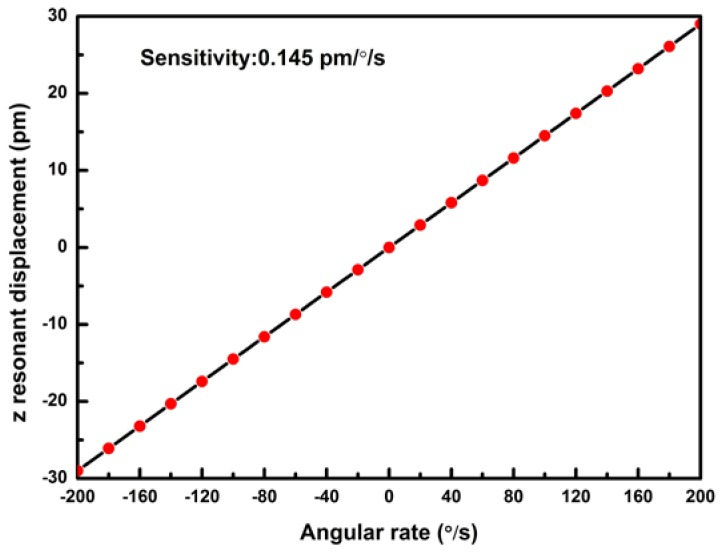
Theoretical sensitivity of the gyroscope—the resonant displacement of *z*-axis vs. angular rate Ω.

The quality factors of this gyroscope are assumed to be *Q*_drive_ = 3000 and *Q*_sense_ = 3000, respectively. The maximum resonant displacement *z_res_*(*L*,$\frac{n\pi }{\omega }$) is shown in Equation (8). Therefore, the sensitivity of this gyroscope is 0.145 pm/◦/s, as shown in Figure 7.

(8) $$z_{res}(L,\frac{n\pi }{\omega })=Q_{\mathrm{drive}}Q_{\mathrm{sense}}z(L,\frac{n\pi }{\omega })=1.45\times 10^{-13}\;\Omega$$

## 4. Conclusions

The AlN-Si composite cantilever gyroscope is based on the novel electrode design and working principles. Because of the piezoelectric effect of AlN, an in-plane movement has been stimulated by two inverse voltages. The AlN-Si composite cantilever gyroscope has been designed with 87.422 kHz drive frequency and 87.414 kHz sense frequency. The mode-matching has been realized by optimizing the width of the cantilever. This gyroscope has a small size, simple structure, and lower requirements for the processing of AlN film. Hence, this cantilever gyroscope shows great potential in the piezoelectric research and consumer electronics fields. 

## Figures and Tables

**Table 1:** Parameters of aluminum nitride (AlN)-Si composite cantilever for calculation and simulation.

Names	Parameters	Values	Units
*E*_AlN_	Young’s modulus	410	GPa
*E*_Si_	Young’s modulus	160	GPa
*E*_Mo_	Young’s modulus	312	GPa
*E*_Al_	Young’s modulus	70	GPa
*d*_AlN_	Thickness	1.5	μm
*d*_Si_	Thickness	20	μm
*d*_Mo_	Thickness	0.3	μm
*d*_Al_	Thickness	0.3	μm
*d*_31_	Piezoelectric coefficient	−2.6	pm/V
*L*	Length of cantilever	600	μm
*W*	Width of cantilever	22.87	μm
*W_d_*_ri_	Width of drive electrode	3	μm
*U*_0_	Amplitude	1	V

**Table 2:** Sizes of AlN-Si composite cantilever gyroscope.

Names	Parameters	Values	Units
*L*	Length of cantilever	600	μm
*W*	Width of cantilever	22.87	μm
*W*_dri_	Width of drive electrode	3	μm
*W*_dri_det_	Width of drive-detected electrode	3	μm
*W*_sen_	Width of sense electrode	4	μm
*d*_AlN_	Thickness	1.5	μm
*d*_Si_	Thickness	20	μm
*d*_Mo_	Thickness	0.3	μm
*d*_Al_	Thickness	0.3	μm
